# Remarks on the Manner in Which the Opening in the Skull Caused by the Trephine, or Other Loss of Bone, Is Filled up

**Published:** 1841-07

**Authors:** 


Art. VII.—*Bemerkungen über die Weise wie die Oeffnung in dem Schädel nach der Trepanation, oder anderem Knochenverlust, ausgefüllt wird.* Von Dr. G. Vrolik, Professor am Athenæum zu Amsterdam, n. a.—*Amsterdam*, 1837. 4to, pp. 18, Ta. 1.

*Remarks on the manner in which the Opening in the Skull caused by the Trephine, or other loss of Bone, is filled up.* By Dr. G. Vrolik, Professor at the Athenæum at Amsterdam, &c.—*Amsterdam*, 1837.

The extent to which apertures in the bones of the skull are repaired, and their mode of reparation, are confessedly among the most obscure subjects of surgical pathology. The remarkable case by Scarpa* is the only one with which we are acquainted in which the reparation was complete; but neither this, nor the numerous cases recorded in which the repair was partial, throw much light on the mode in which the new bone is produced. In this respect the history of the case related in the present memoir has considerable interest.

A Danish sailor fell from the rigging to the deck of his ship upon his forehead, and was carried senseless to the hospital. The late R. Harmss, the surgeon, found the middle of the frontal bone so broken and depressed, that it appeared impossible to elevate the fractured portions without applying the trephine. This he therefore did in two places just above the fracture, and having raised and removed the depressed bones, the patient's consciousness was presently restored. He went on well for the first four weeks, but then became sleepy and then comatose, and died in the sixth week after the accident. On examination, the author says,

"I found the great irregular space from which the portions of bone were removed, closed up by a peculiar tissue, which I believe must be considered as a product of the proper action of the vessels. On looking at the inner table of the skull, I was easily convinced that the dura mater had been so completely separated from it that even the edges of the bone next to the aperture had been deprived of it. The outer table need scarcely be mentioned; the former loss of bone in the space now filled up proves that the tissue occupying it could not have been formed from the periosteum; a conclusion which might also be drawn from the fact of the edge of the wound being quite deprived of that membrane."

From this evidence of the complete want of connexion between the reparative tissue and both the dura mater and periosteum, Dr. Vrolik concludes that it must have been produced from the edges of the opening

* De Anatome et Pathologia Ossium Comm.

in the bone, and points out its relation to the membranes on either side of it as being similar to that of the originally cartilaginous cranium. In this respect, indeed, the similarity is greater than the author has pointed out; for in the ossification of the fœtal skull, there is not first formed a true cartilaginous groundwork for all the flat bones; but, in the growth and ossification of each, the cartilage is formed just precedently to the formation of bone; and thus, as Muscher has shown, the border of each growing bone is, as it were, fringed with cartilage. And the same has happened in the case here described; little processes of bone have shot from all the borders of the aperture in the skull into the new cartilaginous tissue by which it is nearly filled up. But besides this growth of bone from the edges, the plate represents several small osseous patches formed, either in the very middle of the new cartilaginous tissue, or just by the borders of the new bone, which, as the author has well observed, exactly resemble, both in their form and their arrangement, the additional nuclei of bone which are commonly produced in such numbers in the expanded fontanelles of hydrocephalic heads, and of which more perfect examples are found in the ordinary ossa Wormiana.

On the whole, the case here related affords very strong evidence of the similarity between the process of repair of losses of substance in the bones of the skull, and that of their original growth. In both, the growth of bone appears to take place from the edges of that already existing, and by the development and increase of osseous *insular* nuclei in the tissue intermediate between the bones. In both also there is remarkably exhibited that increase by the shooting out of bony rays, of which the most familiar example is presented by the fœtal parietal bone. In the present case, as well as in all the others that have been related, the production of bone by the edges of the trephine-hole, though taking place in exactly the same manner, is far less abundant than that from the edges of the merely fractured portions.

